# Effects of pentoxifylline on canine platelet aggregation

**DOI:** 10.1002/vms3.595

**Published:** 2021-08-06

**Authors:** John M. Thomason, Todd M. Archer, Robert W. Wills, Andrew J. Mackin

**Affiliations:** ^1^ Department of Clinical Sciences Mississippi State University College of Veterinary Medicine Mississippi State Mississippi; ^2^ Department of Comparative Biomedical Sciences Mississippi State University College of Veterinary Medicine Mississippi State Mississippi

**Keywords:** dog, pentoxifylline, platelet

## Abstract

**Background:**

Pentoxifylline can decrease platelet function in humans, but the anti‐platelet effects of pentoxifylline in dogs is unknown. The addition of a luciferin–luciferase reagent during platelet aggregometry can induce a dose‐dependent potentiation of platelet aggregation.

**Objective:**

To determine if exposure to pentoxifylline, without the addition of a luciferin–luciferase reagent during aggregometry, causes canine platelet dysfunction. Our hypotheses were that pentoxifylline would inhibit platelet function, and that the addition of a luciferin–luciferase reagent would obscure detection of pentoxifylline‐induced platelet dysfunction as measured via aggregometry.

**Methods:**

Seven healthy Walker hound dogs. Platelet‐rich plasma (PRP) and whole blood were treated for 30 minutes with pentoxifylline: 0 (control), 1 and 2 μg/mL. The platelet aggregation was determined using optical (maximum amplitude) and impedance (ohms) aggregometry using collagen as the agonists, with and without a luciferin–luciferase reagent. Four samples were analysed per concentration and the results were averaged.

**Results:**

Based on optical aggregometry, there was no difference (*p* = 0.964) in the mean maximum amplitude at any pentoxifylline concentration, with and without the luciferin–luciferase reagent. During impedance aggregometry, the addition of a luciferin–luciferase reagent was associated with significantly (*p* < 0.001) greater platelet aggregation in response to a collagen agonist, regardless of the presence or absence of pentoxifylline.

**Conclusions:**

Pentoxifylline does not exert an in vitro anti‐platelet effect on canine platelet aggregation when collagen is used as an agonist, but it is unknown if long‐term oral drug administration will inhibit platelet aggregation. The addition of a luciferin–luciferase reagent during platelet aggregometry can artificially enhance canine platelet aggregation.

## Graphical abstract

Pentoxifylline does not exert an in vitro anti‐platelet effect on canine platelet aggregation, but is unknown if long‐term oral drug administration will inhibit platelet aggregation. The addition of a luciferin–luciferase reagent during platelet aggregometry can artificially enhance canine platelet aggregation.

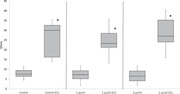

## 1 INTRODUCTION

Pentoxifylline is a methyxanthine derivative that possesses both immunologic and hematologic properties. In dogs, it is most commonly used to treat dermatologic syndromes such as vasculitis and familial dermatomyositis, but in humans it has also been used to treat endotoxemia, cancer and various hypercoagulable states (Marsella et al., 2000; Rees et al., 2003). In addition to its anti‐inflammatory properties, pentoxifylline is also capable of influencing hemostasis (Rees et al., 2003). In humans, as measured by optical aggregometry (using platelet‐rich plasma) and impedance aggregometry (using whole blood), platelet aggregation is significantly decreased during in vitro and in vivo pentoxifylline treatment (De La Cruz et al., 1993; Magnusson et al., 2008).

The cyclic nucleotides, cyclic adenosine monophosphate (cAMP) and cyclic guanosine monophosphate (cGMP), are essential inhibitory intraplatelet second messengers that can interfere with several aspects of platelet function, including degranulation, fibrinogen receptor activation and platelet membrane rearrangement (Gresele et al., 2011; Thomason et al., 2016). Increased levels of platelet cAMP and cGMP will inhibit platelet activation, and increased levels of these nucleotides can be attained either by the binding of endogenous platelet inhibitors (prostacyclin and nitric oxide) to transmembrane receptors or by preventing their breakdown. Phosphodiesterases (PDEs) are enzymes that inactivate cAMP and cGMP, and inhibitors of PDEs can therefore lead to increased levels of intra‐platelet cAMP and cGMP, and subsequent platelet inhibition. PDEs are classified by their affinity and rate of degradation of cyclic nucleotides. Platelets express PDE2 and PDE3, which hydrolyse cAMP, and PDE5, which hydrolyses cGMP. Canine platelet function has previously been shown to be inhibited by a range of phosphodiesterase inhibitors (Boudreaux et al., 1986; Tsien et al., 1982). Pentoxifylline is known to be a non‐selective inhibitor of PDEs (Ueno et al., 2011).

A previous study by Rees and others performed in dogs showed that a single dose of pentoxifylline did not detectably inhibit platelet aggregation using optical aggregometry (Rees et al., 2003). However, that study also incorporated a luciferin–luciferase reagent during aggregometry as an additional indicator of platelet function. Luciferin–luciferase preparations can be added to samples during aggregometry to provide an assessment of platelet activation by evaluating dense granule release from platelets by measuring the luminescence of ATP secretion. Unlike other species, the addition of this reagent during platelet aggregometry in dogs can induce spontaneous, irreversible platelet aggregation (Mehta et al., 1983). In fact, in dogs with hereditary platelet function defects that exhibit weak platelet aggregation during aggregometry, the addition of the luciferin–luciferase reagent causes a dose‐dependent potentiation of platelet aggregation (Callan et al., 1998). In the previous study by Rees and others (Rees et al., 2003), it is possible that the addition of the luciferin–luciferase reagent obscured pentoxifylline‐induced inhibition of platelet function, a phenomenon that we have previously noted in our laboratory when assessing aspirin‐associated platelet dysfunction,^1^ leading to the erroneous appearance of normal platelet aggregation despite the presence of pentoxifylline.

The objective of our study was to determine if exposure to pentoxifylline, without the addition of a luciferin–luciferase reagent during aggregometry, causes dysfunction in canine platelets. Our hypotheses were that pentoxifylline would inhibit canine platelet function by reducing platelet aggregation, and that the addition of a luciferin–luciferase reagent would obscure detection of pentoxifylline‐induced platelet dysfunction as measured via aggregometry.

## 2 MATERIAL AND METHODS

### 2.1 Study population

Blood from seven healthy Walker hound dogs (5 intact females and 2 castrated males) was used for the study. The mean age of the dogs was 4.7 years (range, 2.5–10.5 years). The dogs had not received any medications for at least 2 weeks prior to or during the study. Normal health status was established via physical examination, complete blood count and serum chemistry analysis. Animal use was approved by the Mississippi State University Institutional Animal Care and Use Committee and was in compliance with the requirements of a facility accredited by the American Association for Accreditation of Laboratory Animal Care.

### 2.2 Sample collection and preparation

Blood samples were collected via jugular venipuncture with a 20‐gauge needle into a 4.5 mL vacutainer tube containing 3.2% sodium citrate anticoagulant.^2^ Using a previously published technique (Haines et al., 2016), platelet‐rich plasma (PRP) was created from whole blood via centrifugation. Briefly, whole blood was centrifuged at 1200 × *g* at room temperature for 3 minutes, the PRP supernatant was removed, and the remaining blood sample was centrifuged 1800 × *g* at room temperature for 8 minutes to create platelet‐poor plasma (PPP).

### 2.3 Pentoxifylline incubation

Previously published protocols (Kornreich et al., 2010; Shipley et al., 2013) were modified to evaluate canine platelet function via aggregometry following the in vitro exposure of PRP to two different concentrations of pentoxifylline,^3^ plus a saline control. Exposure concentrations were selected based on approximate plasma concentrations of pentoxifylline attained in dogs with standard oral dose rates (Marsella et al., 2000). Briefly, two concentrations of pentoxifylline working solution, 1  and 2 μg/mL, were created from a stock solution containing 10 mg/mL of pentoxifylline in 0.9% sodium chloride. To determine the dose effect on platelet aggregation, 10 μL from each working solution was added to 990 μL of whole blood, inverted three times and incubated, at room temperature, for 30 minutes. For the control sample, 10 μL of 0.9% sodium chloride was added to the PRP and whole blood, respectively. Following incubation, the treated samples were transferred to a cuvette for optical and impedance aggregometry analysis.

### 2.4 Platelet aggregometry

#### 2.4.1 Optical aggregometry

A 2‐channel optical platelet aggregometer^4^ was used to analyse platelet aggregation. Aggregation was assessed using collagen^5^ (10 μg/mL) as the agonist, with a temperature of 37°C, and a stirring speed of 1200 rpm. Prior to the study, the collagen concentration was optimised to achieve consistent platelet activation. Samples were analysed based on the manufacturer's standard guidelines.^6^ Briefly, 450 μL of pentoxifylline‐exposed PRP was placed into a glass cuvette with a stir bar, and 500 μL of PPP was placed into a cuvette without a stir bar. Samples were incubated for 1 minute at 37°C, placed into the aggregometer, and stable baseline values corresponding to 0% and 100% aggregation were obtained using PRP and PPP, respectively. Collagen was added to the PRP, and platelet aggregation was monitored for 8 minutes. The maximal percentage aggregation was calculated and recorded using computer software.^7^ For each pentoxifylline concentration, four total samples were analysed, and the results were averaged to yield a single value. For the samples containing the luciferin–luciferase reagent,^8^ 50 μL (8 μg luciferin, 880 Units d‐luciferase) was added to the PRP prior to the addition of collagen. All samples were analysed within 4 hours of blood collection. Based on recommendations published by the International Society of Thrombosis and Haemostasis Platelet Physiology and Scientific and Standardization Committee, the platelet count in the PRP was not adjusted to a standardised count by dilution with PPP prior to analysis (Cattaneo et al., 2007; Linnemann et al., 2008; Mani, 2005).

#### 2.4.2 Impedance aggregometry

The same aggregometer used for optical analysis was converted for use for impedance analysis, and samples were analysed according to the manufacturer's standard guidelines. Briefly, 450 μL of 0.9% sodium chloride and 450 μL of pentoxifylline‐exposed whole blood were transferred to a plastic cuvette containing a magnetic stir bar. Samples were incubated at 37°C for 5 minutes, placed into the aggregometer, and a reusable impedance probe was inserted into each cuvette. Collagen, 10 μg/mL, was then added to the sample and aggregation was monitored for 12 minutes. The maximal amplitude, measured in ohms, was calculated and recorded as an indicator of maximal aggregation. Four samples per pentoxifylline concentration were analysed, and the results were averaged to yield a single value. For the samples containing luciferin–luciferase, 100 μL (16 μg luciferin, 1760 Units d‐luciferase) was added to the whole blood prior to the addition of collagen. All samples were analysed within 4 hours of blood collection.

### 2.5 Statistical analysis

Based on the mean and standard deviation values (53.8% ± 9.9) for optical aggregometry from a previous study (Mclewee et al., 2018), with a power of 90% and significant level of 0.05, 6 dogs were calculated to be needed to identify a 25% decrease in platelet aggregation. Separate linear mixed models using PROC MIXED were fit for median optical and median impedance aggregometry outcome in a statistical computer program.^9^ Pentoxifylline concentration, inclusion of luciferin–luciferase, and pentoxifylline concentration by luciferin–luciferase interaction were included as fixed effects. Dog identification was included as a random effect with variance components covariance structure. Differences in least squares means were determined for outcomes with significant main effect or interaction terms. The distribution of the conditional residuals was evaluated for each outcome to ensure the assumptions of normality and homoscedasticity for the statistical method had been met. An alpha level of 0.05 was used to determine statistical significance for all methods.

## 3 RESULTS

### 3.1 Optical aggregometry

**FIGURE 1:**
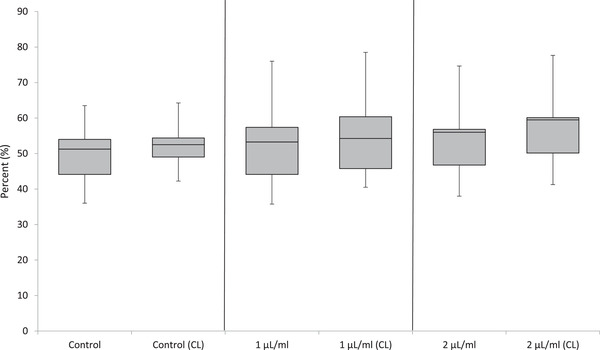
Maximum amplitude (percentage) of aggregation via optical aggregometry in canine blood exposed to saline control (0.9% sodium chloride) or pentoxifylline (1 and 2 μL/mL), both with and without concurrent exposure to luciferin–luciferase [Chronolume^®^ (CL)]. The box and whiskers plot demonstrates the median (line), interquartile range (box) and total range (whiskers)

The optical aggregometry results for all pentoxifylline concentrations are represented in Figure 1. There were no significant differences in the maximum amplitude at any pentoxifylline concentration (*p* = 0.964). The addition of luciferin–luciferase also had no significant effect on aggregometry results.

### 3.2 Impedance aggregometry

**FIGURE 2:**
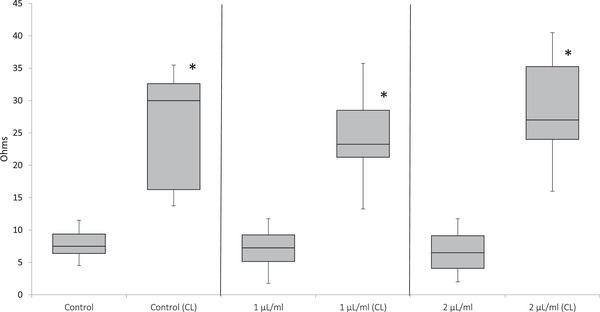
Maximum amplitude (ohms) of aggregation via impedance aggregometry in canine blood exposed to saline control (0.9% sodium chloride) or pentoxifylline (1 and 2 μL/mL), both with and without concurrent exposure to luciferin–luciferase [Chronolume^®^ (CL)]. The box and whiskers plot demonstrates the median (line), interquartile range (box) and total range (whiskers). ‘*’ illustrate significant differences from non‐Chronolume^®^ values

The impedance aggregometry results for all pentoxifylline concentrations are represented in Figure 2. The addition of a luciferin–luciferase reagent was associated with significantly (*p* < 0.001) greater platelet aggregation in response to a collagen agonist, regardless of the presence or absence of pentoxifylline.

## 4 DISCUSSION

The results of our study indicate that, contrary to our hypothesis, the in vitro exposure of platelets to pentoxifylline does not inhibit platelet aggregation in dogs when using collagen as an agonist. Our results are in contrast to similar studies using human platelets, where exposure to pentoxifylline in vitro inhibited platelet aggregation (De La Cruz et al., 1993; Magnusson et al., 2008). In contrast, a previous ex vivo study performed in dogs treated with pentoxifylline did not detect drug‐associated inhibition of platelet aggregation, a finding that is now supported by the findings of our in vitro study (Rees et al., 2003).

Our study evaluated the effects of pentoxifylline on platelet function both with and without the use of a luciferin–luciferase reagent, a reagent that can artificially enhance platelet aggregation and potentially obscure any pentoxifylline‐associated inhibition of platelet function. Luciferin–luciferase preparations allow for additional assessment of platelet function by evaluating dense granule release following platelet activation by measuring the luminescence of ATP secretion. Measurement of luminescence provides a more direct measure of platelet activation compared to light scattering properties associated with platelet aggregation within a PRP sample, and it is common laboratory practice to utilise aggregometers that concurrently measure both aggregation and luminescence following the addition of a platelet agonist.

The luciferin–luciferase preparation used in our study, and a previous ex vivo pentoxifylline study in dogs (Rees et al., 2003), however, has been shown to potentiate platelet aggregation, as evaluated by optical aggregometry, in both normal dogs and thrombocytopathic dogs when stimulated with ADP, collagen and thrombin, suggesting that the use of this product in dogs could obscure congenital and acquire thrombocytopathic conditions in dogs (Callan et al., 1998). In fact, previous work in our laboratory has confirmed that the addition of luciferin–luciferase can artefactually obscure aspirin‐associated inhibition of platelet aggregation.^1^ The reason this luciferin–luciferase preparation appears to induce platelet activation in dogs, but not humans, is unknown, but the in vitro addition of magnesium sulfate, an ingredient in the luciferin–luciferase product, prior to analysis has been proposed as a potential mechanism (Callan et al., 1998). Our study confirmed that the presence of luciferin–luciferase significantly increased the magnitude of platelet aggregation in response to a collagen agonist, independent of the presence of pentoxifylline. Interestingly, this effect was only observed with impedance aggregometry, and not with optical aggregometry. The reason for the difference in the platelet responses to luciferin–luciferase with the two different types of aggregometry is unclear. Nevertheless, our study confirmed the findings of the previous study by Rees and others (Rees et al., 2003), that pentoxifylline does not appear to inhibit canine platelet aggregation when using collagen as an agonist, and that this observation is not erroneously affected by the presence of luciferin–luciferase.

In both human and veterinary medicine, platelet aggregometry is considered the gold standard for evaluation of platelet function (Lordkipanidzé et al., 2009; Nielsen et al., 2007). Aggregometry measures the ability of platelets to aggregate following the use of specific agonists to activate platelets in either PRP or whole blood (Lordkipanidzé et al., 2009). Similar to previous studies (Rees et al., 2003), we used collagen as the agonist to activate the platelets. Although collagen provides consistent platelet activation and is commonly used to assess drug‐induced platelet dysfunction, a panel of different agonists, including ADP, thrombin, epinephrine and arachidonic acid, could have provided additional assessment of potential pentoxifylline‐associated platelet dysfunction and, if dysfunction was detected, unveiled potential mechanisms for this effect. Using a similar protocol as Rees et al., our used collagen as the only agonist to assess the effects of pentoxifylline on canine platelet function. Additionally, other studies that evaluated the effects of pentoxifylline on platelet function in humans demonstrated that pentoxifylline had a greater inhibitory effect on platelet function when collagen was used as an agonist compared to adrenaline and arachidonic acid (De La Cruz et al., 1993). Because our study indicated that pentoxifylline did not have an effect on platelet aggregation when collagen was used as an agonist, we do not believe that the use of adrenaline and arachidonic acid as agonists would have provided a different effect on platelet function. Finally, both optical and impedance aggregometry evaluate platelet aggregation under low shear forces, and the addition of an analyser that evaluated platelet function under high shear forces could have provided an additional assessment of potential pentoxifylline‐associated platelet dysfunction.

Compared to the control samples, the PRP and whole blood samples that contained the luciferin–luciferase reagent had an extra 50 and 100 μL of volume, respectively. The biggest effect of this additional volume would have been a dilutional effect on the ability of platelets to aggregate. In order to aggregate, platelets need to be in close contact and the additional volume in the luciferin–luciferase samples could have separated the platelets, making it more difficult to aggregate. However, if there was a dilutional effect in our study, it does not appear to have a major impact of platelet aggregation because the samples that contained the luciferin–luciferase reagent had similar or stronger platelet aggregation compared to the samples without the additional volume.

Although pentoxifylline has not been shown to inhibit platelet function in dogs, other PDE inhibitors have been associated with drug‐induced platelet dysfunction. Dipyridamole, for example, inhibits both PDE3 and PDE5, and may potentiate the inhibitory effects of prostacyclin and nitric oxide (Gresele et al., 2011). Dipyridamole is an ineffective inhibitor of platelet function when used as a single agent anti‐platelet therapy in dogs, but enhances platelet dysfunction when combined with low dose aspirin (Weselcouch et al., 1987). Pimbobendan, another inhibitor of PDE3 and PDE5 isoforms, does not inhibit platelet function at clinically applicable doses in dogs (Shipley et al., 2013). Sildenafil also inhibits PDE5 but, in humans, does not inhibit platelet aggregation when used as a single agent (Gresele et al., 2011; Wallis et al., 1999). The potential inhibitory effects of pimobendan or sildenafil when combined with an antiplatelet agent in dogs are unknown, and similarly it is also unknown if the combination of pentoxifylline with other anti‐platelet agents will enhance inhibition of canine platelet function.

Our study had several limitations. First, this study was conducted in vitro, and not ex vivo with aggregometry performed on samples collected from dogs receiving pentoxifylline. The ongoing administration of pentoxifylline to dogs, especially over an extended period of time, may have an accumulative anti‐platelet effect that was not detected in our study. Additionally, an ex vivo study conducted on samples from dogs administered pentoxifylline would have the potential to determine the combined effects of pentoxifylline and its metabolites, rather than the effects of pentoxifylline alone. Interestingly, while Rees et al. found that administration of pentoxifylline to healthy dogs did not inhibit ex vivo platelet aggregation, in the same study the authors detected pentoxifylline metabolites comparable to those which, with human platelets, have been shown to have platelet inhibitory effects (Magnusson et al., 2008). The reasons for the apparent differences between the responses of canine platelets and the responses of human platelets to pentoxifylline and its metabolites are unclear. Second, although a pre‐study sample size calculation indicated that seven dogs would be appropriate to detect a 25% decrease in platelet aggregation, a larger population of animals would have allowed for detection of more subtle differences. Aggregometry results in our study, however, were remarkably consistent regardless of the presence or absence of exposure to pentoxifylline, suggesting that, even if subtle drug‐induced effects do exist, they would be unlikely to be sufficient to have biological significance. Third, all the dogs in our study were of the same breed, Walker hounds, and it is possible that inclusion of other breeds of dogs would have provided different results. Fourth, our study only evaluated two concentrations of pentoxifylline, based on anticipated plasma concentration in dogs receiving oral pentoxifylline (Marsella et al., 2000). Exposure of platelets to higher concentrations of pentoxifylline, although such concentrations would be unlikely to be clinically relevant, may have revealed concentration‐dependent alterations in platelet aggregation. Fifth, the user manual for the aggregometer used in this study recommends performing platelet analysis within 3 hours of venipuncture. In our study, a majority of samples were analysed within 3 hours of venipuncture, but with the addition of a pentoxifylline incubation period prior to analysis, a few samples were analysed between 3 and 4 hours after venipuncture. It is possible that the extended period of time could have adversely affected our results, although this slightly extended time period has been used previously in dogs (Blois et al., 2010). Finally, the samples in our study were exposed to pentoxifylline for only a single fixed time duration of 30 minutes. This exposure duration was selected to coincide with peak plasma concentrations since, when given with food, peak plasma concentrations of pentoxifylline occur 30 minutes after the initial oral administration (Marsella et al., 2000). It is possible that with a longer in vitro platelet exposure to pentoxifylline, the results of our study would have been different.

The results of our study confirm that, unlike in humans, pentoxifylline does not exert an in vitro inhibitory effect on canine platelet aggregation using collagen as an agonist. Our study also confirms that the addition of a luciferin–luciferase reagent during platelet aggregometry can artificially enhance platelet aggregation, which could potentially have an impact on interpretation of studies performed using this reagent. Our study mimics the effects on platelet function of a single dose exposure to pentoxifylline, and it is still unknown if long‐term oral pentoxifylline administration will inhibit platelet aggregation in dogs.

## CONFLICT OF INTEREST

The authors have no known conflicts of interest to disclose.

## ETHICS STATEMENT

The authors confirm that the ethical policies of the journal, as noted on the journal's author guidelines page, have been adhered to and the appropriate ethical review committee approval has been received. The US National Research Council's guidelines for the Care and Use of Laboratory Animals were followed.

### PEER REVIEW

The peer review history for this article is available at https://publons.com/publon/10.1002/vms3.595.

## Footnotes

1 Haines J. In vitro and In vivo Assessment of Platelet Function in Healthy Dogs During Low‐Dose Aspirin Therapy. Masters of Veterinary Medicine, Thesis. 2014. Mississippi State University College of Veterinary Medicine.

2 3.2% sodium citrate, Vacutainer tube, Becton Dickinson, Franklin Lakes, NJ.

3 Pentoxifylline, Sigma Aldrich, St. Louis, MO.

4 Chronolog 700 Whole Blood/Optical Lumi‐Aggregometer, Chronolog Corporation Haverton, PA.

5 Collagen, Chronolog Corporation, Haverton, PA.

6 Chronolog 700 Manual, Chronolog Corporation, Haverton, PA.

7 Chronolume^®^, Chronolog Corporation, Haverton, PA

8 AGGRO/LINK 8, Chronolog Corporation, Haverton, PA.

9 SAS for Windows 9.4 SAS Institute, Inc., Cary, NC, USA.
